# Clomiphene citrate mild stimulation improved follicular development outcomes in PCOS women with high luteinizing hormone and poor ovarian response: A case report

**DOI:** 10.1097/MD.0000000000031323

**Published:** 2022-10-21

**Authors:** Xiaojing Yang, Meiyan Jiang, Miao Deng, Huanhuan Zhang, Zhenyun Lin, Xiaoyang Fei, Hongyan Zhang

**Affiliations:** a Department of Reproductive Medicine Center, Hangzhou Women’s Hospital, Hangzhou, Zhejiang, China.

**Keywords:** Clomiphene citrate, in vitro fertilization, luteinzing hormone, polycystic ovarian syndrome, poor ovarian response

## Abstract

Polycystic ovary syndrome (PCOS) is a main cause of anovulatory infertility in women of reproductive age. About 30% to 50% of patients with PCOS has high serum basal luteinizing hormone (LH) levels, and almost 5% of PCOS women with high LH have poor ovarian response (POR). We reported a case of a PCOS woman with high basal LH levels who canceled due to POR during two consecutive controlled ovarian stimulation treatments, which was considered to be related to the suppression of LH levels during downregulation. Clomiphene citrate (CC) combined with human menopausal urinary gonadotropin (HMG) mild regimen did not affect LH levels and obtained good follicular development, providing a new treatment insight for patients with PCOS combined with POR.

**Patient concerns:**

A 28-year-old PCOS woman with high basal LH levels, underwent IVF assisted pregnancy treatment in our hospital, whom canceled due to POR during two traditional controlled ovulation induction program. Follicular development was finally achieved with CC milder protocol.

**Diagnosis:**

This patient with the diagnosis of PCOS was undergone IVF assisted pregnancy treatment in our hospital.

**Interventions:**

CC protocol supports the development of follicular.

**Outcomes:**

CC protocol resulted in better follicular development and high-quality embryos due to the continuous maintenance of an elevated LH levels.

**Conclusion:**

PCOS women with poor ovarian response required relatively higher LH to maintain the normal development of follicles.

## 1. Introduction

Polycystic ovary syndrome (PCOS) is currently considered the leading cause of anovulatory infertility in reproductive-age women, affecting up to 15% of this population worldwide.^[1]^ It is characterized by dysregulated pulses of luteinizing hormone (LH), ovulatory dysfunction, high androgen level, and insulin resistance.^[2]^ Elevated LH concentrations observed in up to 75% of women with PCOS, measured at a single time point above 95% of normal.^[3]^ During the treatment process of in vitro fertilization-embryo transfer (IVF-ET), PCOS often manifests as increased or decreased ovarian response, prone to premature luteinization of follicles, resulting in impaired oocyte and embryo quality. It is still controversial whether basal high LH levels cause oocyte quality damage during controlled ovulation.

Previous studies have suggested that high LH levels in follicular phase will negatively affect the quality of oocytes in PCOS patients, thereby affecting the rate of fertilization and clinical pregnancy.^[4]^ However, recent studies have shown that high LH levels do not impair the normal development of oocytes in PCOS patients.^[5]^

We reported a case of PCOS woman with high basal LH levels who canceled due to poor ovarian response (POR) during two consecutive controlled ovarian stimulation treatments. Follicular development was finally achieved with clomiphene citrate (CC) milder protocol. Different from the traditional controlled ovulation induction program, CC milder protocol did not down-regulate the pituitary gland and affect the serum LH level, thus the patient’s serum LH continued to maintain an elevated level. However, the continuous high levels of LH did not adversely affect the quality of oocytes and embryos in PCOS patients. We consider that PCOS patients may require relatively high serum LH levels to maintain normal oocyte development due to differences in neuroendocrine patterns and genetic phenotypic polymorphisms.

## 2. Case presentation

The patient, a 28-year-old woman (weight: 50 kg, body mass index (BMI): 23.13 kg/m^2^), underwent IVF assisted pregnancy treatment in our hospital due to anovulation with the diagnosis of PCOS. This patient had a menstrual cycle of 30 to 90 days and a period duration of 4 to 5 days. The serum anti-mullerian hormone (AMH) level was 7.78 ng/mL, basal follicle stimulating hormone (FSH), LH and estradiol (E2) levels were 6.8 mIU/mL, 13.96 mIU/mL and 40 pg/mL, respectively. Gynecological color Doppler ultrasound showed that more than 15 antral follicles per ovary which suggested multiple cystic changes in the bilateral ovaries. The patient had previously been treated with 2 cycles of ovulation stimulation and 3 cycles of artificial insemination without pregnancy.

## 3. Interventions and outcomes

**Figure 1.**
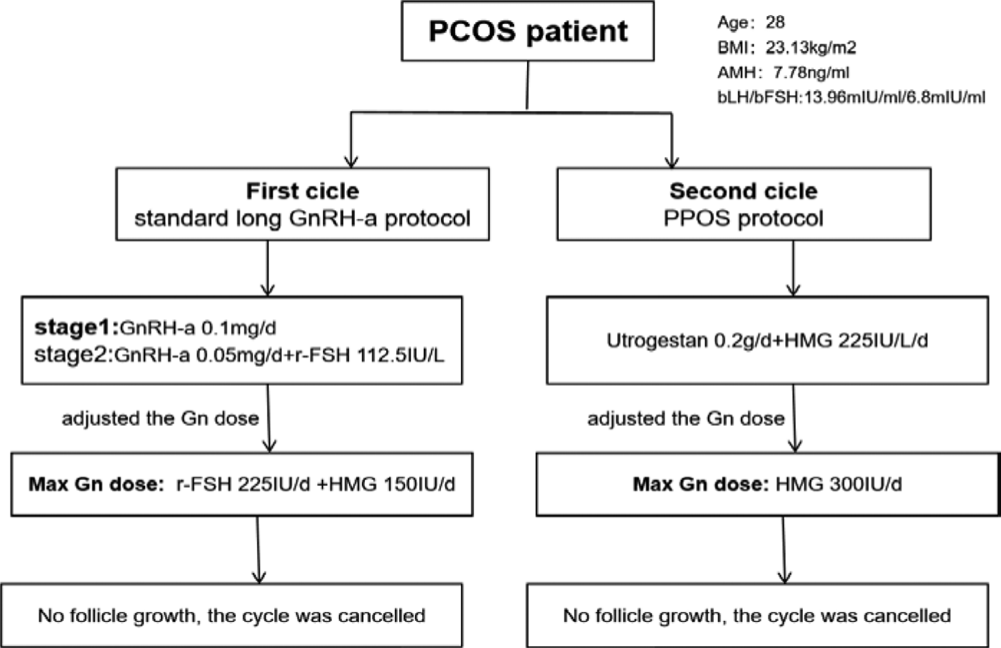
Controlled ovulation protocol in the 1st and 2nd cycle. In the first cycle: a standard long gonadotrophin releasing hormone agonist (GnRH-a) protocol. In the second cycle: Progestin-primed ovarian stimulation (PPOS).

A standard long gonadotrophin releasing hormone agonist (GnRH-a) protocol was used for the first controlled ovulation induction cycle.^[6]^ For pituitary down-regulation, 0.1 mg of short-acting GnRH-a (Decapeptyl, Ferring, Kiel, Germany) was administered daily starting in the mid-luteal phase. After 14 days, the pituitary reached the standard of downregulation (LH was 1.94 IU/L, E2 was 14.45 pg/mL, endometrial thickness was 5 mm, no functional ovarian cyst). Administration of recombinant FSH (r-FSH, Gonal-F; Merck-Serono, Eysins, Switzerland) for ovarian stimulation was started at an initial dose of 112.5 IU/d. GnRH-a was reduced to 0.5 mg/day. We adjusted the gonadotropin (Gn) dose promptly according to follicle growth and the levels of serum LH, E2 and progesterone every 3 to 5 days. On the 10th day of ovulation induction, the dose Gn was adjusted to 225 IU/d of r-FSH and 150 IU/d of human menopausal urinary Gn (human menopausal urinary gonadotropin (HMG), Lizhu Pharmaceutical Trading Co., China).On the 14th day, ultrasound monitoring showed that the largest follicle diameter was 10 mm, and the cycle was canceled (Fig 1).

In the second cycle, Progestin-primed ovarian stimulation (PPOS) was adopted.^[5]^ A low dose of HMG (150 IU daily), Micronized Progesterone (Besins Manufacturing Belgium, France) 0.2 g daily were started from cycle day 3. Follicle monitoring by transvaginal ultrasound and serum hormone measurements (LH, E2 and progesterone) were performed 5 days later. HMG doses were then adjusted according to the ovarian response (range 150–300 IU daily). On day 12 of ovulation promotion, the largest follicle diameter of both ovaries was 12 mm, and the cycle was canceled again. Two cycles with a poor responder after maximal stimulation are sufficient to classify the patient as a POR^[7]^ (Fig 1).

CC milder stimulation protocol was used in the third cycle. On the 3rd day of menstruation, basal LH, E2 and P were 13.35 mIU/mL, 26 pg/mL and 0.4 pg/mL, respectively, and CC 100 mg/d was started. 7 days later, follicle monitoring by transvaginal ultrasound (5 follicles of 8 mm to 10 mm in diameter in both ovaries) and serum hormone measurements (LH was 13.48 mIU/mL, E2 was 68.99 pg/mL, P was 0.42 pg/mL) were performed, and we started adding HMG 225 IU/d at the same time. HMG doses were then adjusted according to the ovarian response (range 150–300 IU daily). On day 18, there were 3 follicles ≥ 18 mm in diameter and 2 follicle between 14 and 17 mm in diameter in both ovaries. LH was 10.36 mIU/mL, E2 was 2104.95 pg/mL, P was 1.09 pg/mL. Oocyte maturation was triggered by human chorionic Gn 10000IU (HCG, Lizhu Pharmaceutical Trading Co., China). Cumulus oocyte complexes were collected 36 hours later. All follicles larger than 10 mm in diameter were aspirated. Six oocytes were obtained and routine luteal support (micronized progesterone 0.2 g daily) was given after oocyte retrieval. Fertilization was carried out in vitro. Embryos were examined for the number or regularity of blastomeres and the degree of fragmentation. All oocytes were fertilized on the first day after oocyte retrieval, and 3 oocytes had 2 pronuclei, 3 with 3PN. On the 3rd day of fertilization, 3 top-quality cleavage-stage embryos (8CII, 6CII*2) were obtained according to the Gardner’s criteria,^[8,9]^ then were frozen and preserved by vitrification (Table 1). This patient has signed the consent form for publication of this case which was approved by the Ethics Committee of Hangzhou Women’s Hospital.

**Table 1:** CC milder stimulation protocol was used in the third cycle.

Date/Menstrual cycle	7.29/3	8.5	8.9	8.12	8.13	8.15
Date of Gn		1	5	8	9	
Folliclediameter of right ovary (mm)	5 × 10 + F	9.5 × 2F	11 × 1F	16	19	
			10.5 × 1F			
				11	13	
			8 × 1F			
Folliclediameter of left ovary (mm)	5 × 10 + F	8.5 × 3F	10.5	17	20	
				13.5	18	
					16.5	
				13 × 2F	15	
				11.5 × 2F		
			10 × 2F			
					13	
Endometrium (mm)	4	4.5	5	5.5	6	
CC (mg)	100	100	100	100	100	
HMG (IU)		225	225	300	300	
HCG (IU)					10000	
E2 (pg/mL)	26	68.99	582.26	1595	2104.95	OPU
LH (mIU/mL)	13.35	13.48	14.02	13.48	10.36	
P (ng/mL)	0.4	0.42	0.79	1.7	1.09	

CC = clomiphene citrate, E2 = estradiol, HMG = human menopausal urinary gonadotropin, LH = luteinizing hormone, P = progesterone.

## 4. Discussion

PCOS is a complex endocrine metabolic disorder caused by dysfunction of the hypothalamic-pituitary-ovarian (H-P-O) axis. A large epidemiological study has shown that the incidence of women with PCOS is 5.6% in the Chinese Han population.^[10]^ Endocrine imbalances include elevated total testosterone (TT) levels, high luteinizing hormone/follicle stimulating hormone ratio (LH:FSH) along with metabolic derangements.^[2]^ Previous theories suggest that defects in the H-P-O axis lead to increased LH secretion. The increased frequency and amplitude of pulsatile LH secretion may be related to hyperandrogenemia, altered central GnRH pulse patterns and genetic factors. Increased LH secretion leads to an increase in androstenedione. Androstenedione is first converted to testosterone in the thecal cells via 17β reductase and released into the blood. Secondly, androstenedione can also be converted into E2 by FSH-dependent aromatase enzyme in the granulosa cells of the ovary, which promotes follicular growth and development and ovulation in the early follicles. Normal follicle growth is the result of the complementary action of FSH and LH. LH plays a crucial role in oocyte maturation and induction of ovulation.^[2,3,11]^

Previous studies have shown that increased levels of LH will lead to persistently high levels of androstenedione, further hindering the further development of follicles to mature follicles, leading to ovulation disorders, resulting in premature luteinization of follicles, reducing oocyte quality, and affecting clinical pregnancy rates.^[2]^ Decreased levels of LH may lead to insufficient androgen synthesis, resulting in a relative lack of estrogen production in the intrafollicular environment, delaying oocyte maturation, and resulting in decreased fertilization rates and embryo quality.^[11]^ Therefore, the concept of “LH therapeutic window” was proposed. At present, there are also no clear guidelines on the optimal level of serum LH and timing of its supplementation are fewer in number.^[11]^ Studies have shown that serum LH levels should be between 1.2 IU/L and 5.0 IU/L for optimal follicular development during cycles in which endogenous LH is suppressed.^[12]^

At present, most reproductive centers adopt a standard long GnRH-a protocol for controlled ovulation induction. Which is thought to suppress the secretion of pituitary Gn, inhibit premature of LH surge, avoid premature follicular luteinisation and improve oocyte quality, while facilitating synchronization of follicular development and increasing the number of oocyte gained, thus improving clinical pregnancy rates.^[13]^ Due to the special characteristics of endocrine disorder, most PCOS women have a good ovarian response after controlled ovulation with long GnRH-a protocol. However, almost 5% of PCOS women have POR. POR is a pathological condition in which the ovary responds poorly to Gn stimulation.^[14]^

This case was a young PCOS woman with high basel LH level combined with POR. The first cycle was downregulated with a long GnRH-a protocol, and was canceled due to follicular dysplasia. In the second cycle, the PPOS protocol was used. We found a mild trend of elevated LH in the early stages of drug use. With the negative feedback effect of progestin, endogenous LH was suppressed, and resulted in a decreased LH levels, affected follicle development, thus the cycle was canceled. Ultimately, we succeeded in achieving good follicle development with the CC mild stimulation protocol without pituitary downregulation.

Our study showed that during the treatment of CC protocol, the serum LH was close to the physiological level as the pituitary function was not suppressed. Patients could show elevated levels of LH in the follicular phase. CC was used to inhibit the negative feedback of estrogen, promote the release of FSH and LH from the hypothalamus, and improve the quality of oocytes and embryos. More important, no spontaneous LH surge occurred even with the relatively higher LH. The patient finally achieved an ideal pregnancy outcome. Ye et al^[5]^ showed that high LH levels did not adversely affect the quality of oocyte and embryo. PCOS may require high LH levels to maintain normal oocyte development. This is consistent with the results of our case. Therefore, we believe that high LH in PCOS women does not affect oocyte quality and lead to premature follicle luteinization. Conversely, higher LH levels are required to support oocyte development and ovulation due to altered secretion and regulatory patterns of the H-P-O axis and altered LH conformation and receptors in PCOS patients.

## 5. Conclusion

The CC combined with HMG mild regimen did not affect LH levels and obtained good follicular development, providing a new treatment insight for patients with PCOS combined with POR.

## Acknowledgments

The authors thank all team members for their contributions to the study.

## Author contributions

**Conceptualization:** Hongyan Zhang.

**Data curation:** Huanhuan Zhang, Meiyan Jiang, Miao Deng.

**Writing-original draft:** Hongyan Zhang, Xiaojing Yang.

**Writing-review & editing:** Xiaoyang Fei, Zhenyun Lin,.
